# Naturally Acquired Humoral Immunity Against *Plasmodium falciparum* Malaria

**DOI:** 10.3389/fimmu.2020.594653

**Published:** 2020-10-29

**Authors:** S. Jake Gonzales, Raphael A. Reyes, Ashley E. Braddom, Gayani Batugedara, Sebastiaan Bol, Evelien M. Bunnik

**Affiliations:** Department of Microbiology, Immunology and Molecular Genetics, Long School of Medicine, The University of Texas Health Science Center at San Antonio, San Antonio, TX, United States

**Keywords:** antibody, protection, variant surface antigens, PfEMP1, merozoite, vaccine

## Abstract

Malaria remains a significant contributor to the global burden of disease, with around 40% of the world’s population at risk of *Plasmodium* infections. The development of an effective vaccine against the malaria parasite would mark a breakthrough in the fight to eradicate the disease. Over time, natural infection elicits a robust immune response against the blood stage of the parasite, providing protection against malaria. In recent years, we have gained valuable insight into the mechanisms by which IgG acts to prevent pathology and inhibit parasite replication, as well as the potential role of immunoglobulin M (IgM) in these processes. Here, we discuss recent advances in our understanding of the mechanisms, acquisition, and maintenance of naturally acquired immunity, and the relevance of these discoveries for the development of a potential vaccine against the blood stage of *Plasmodium falciparum*.

## Introduction

Malaria is a deadly disease caused predominantly by the parasite *Plasmodium falciparum*. In 2018, an estimated 228 million cases occurred globally, resulting in 405,000 deaths, of which most were children (1). With the distribution of long-lasting insecticide-treated bed nets, increased insecticide spraying, and earlier diagnosis and treatment, major progress has been made in reducing morbidity and mortality since 2010. However, malaria continues to be a major global public health challenge and have devastating socioeconomical impact, mainly in Sub-Saharan Africa. Because of the spread of drug-resistant parasites and insecticide-resistant mosquitoes, as well as lack of access to treatment, vaccine development remains the most promising avenue for the eradication of malaria.

**Figure 1:**
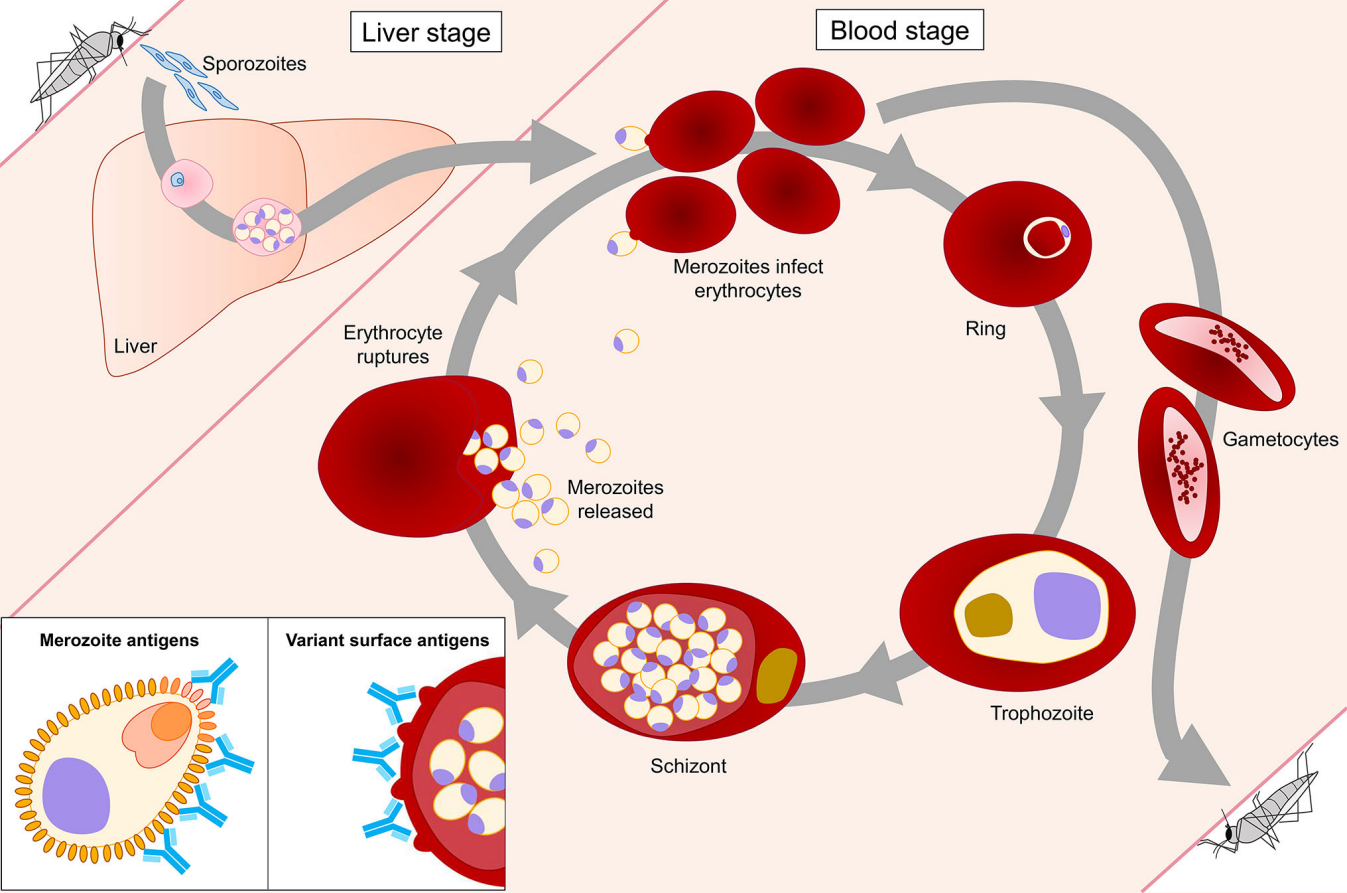
*Plasmodium falciparum* life cycle stages in the human host. The blood stage of *P. falciparum* is the only life cycle stage responsible for disease in the human host. During the intraerythrocytic developmental cycle (IDC), merozoites invade erythrocytes, followed by development and replication of the parasite through ring, trophozoite, and schizont stages, until new merozoites egress and the cycle repeats. Antibody responses that protect against malaria are directed against two classes of antigens expressed during the IDC. Merozoite antigens, such as MSP1 and AMA1, are common targets of antibodies generated during natural *P. falciparum* infection. Antibodies against these antigens prevent merozoite invasion of erythrocytes *via* various effector mechanisms, including neutralization, opsonic phagocytosis, and complement activation. Variant surface antigens (VSAs) are expressed on the surface of infected erythrocytes. Antibodies against VSAs, including PfEMP1, RIFINs, and STEVORs, prevent cytoadherence of infected erythrocytes to vascular endothelium and rosetting, thereby promoting clearance of parasite-infected erythrocytes by the spleen.

Vaccine development efforts have focused on multiple stages of the parasite life cycle, including the pre-erythrocytic stages and the asexual blood stage (**Figure 1**). While the development of sporozoite vaccines is in a more advanced stage than blood stage vaccines [reviewed in (2, 3)], strategies against both stages still face multiple hurdles. Therefore, the ultimate malaria vaccine may need to target both life cycle stages to reach sufficient vaccine efficacy. The *Plasmodium* asexual blood stage is responsible for symptomatic disease and can elicit a robust immune response [reviewed in (4)]. Over the course of multiple infections, antibody responses against blood stage parasites broaden and reach a level that protects against malaria (5–7). However, eliciting a long-lasting, protective antibody response by vaccination has proven a difficult task. Among the many reasons for the failures of historical blood stage vaccine candidates are i) sequence variation in vaccine targets that resulted in parasite strain-specific responses (8, 9), ii) inability of the vaccine to elicit sufficiently high antibody titers necessary for protection (10, 11), and iii) quick waning of elicited immune responses (6, 12–15). In parallel to these obstacles for vaccine development, many questions about the nature of naturally acquired immunity remain. For example, it is not fully understood why immunity against malaria develops relatively slowly, how long naturally acquired antibody responses are maintained in the absence of re-exposure, whether antibody responses are strain-transcending or a combination of strain-specific responses, and which (combinations of) antigen(s) should be prioritized for vaccine development.

The main antigenic targets of blood stage parasites can be divided into two categories: i) parasite variant surface antigens on the cell membrane of infected erythrocytes and ii) proteins that are located on the merozoite surface or secreted by merozoites during erythrocyte invasion (**Figure 1**). Recent studies examining naturally acquired immunity have revealed important insights into antibody responses against these two groups of *P. falciparum* antigens, including their development and maintenance, key molecular and immunological mechanisms of parasite inhibition, and a potential role for immunoglobulin M (IgM) in the protective response. This review provides an overview of these discoveries and highlights their relevance for vaccine development.

### *Plasmodium falciparum* Variant Surface Antigens

**Figure 2:**
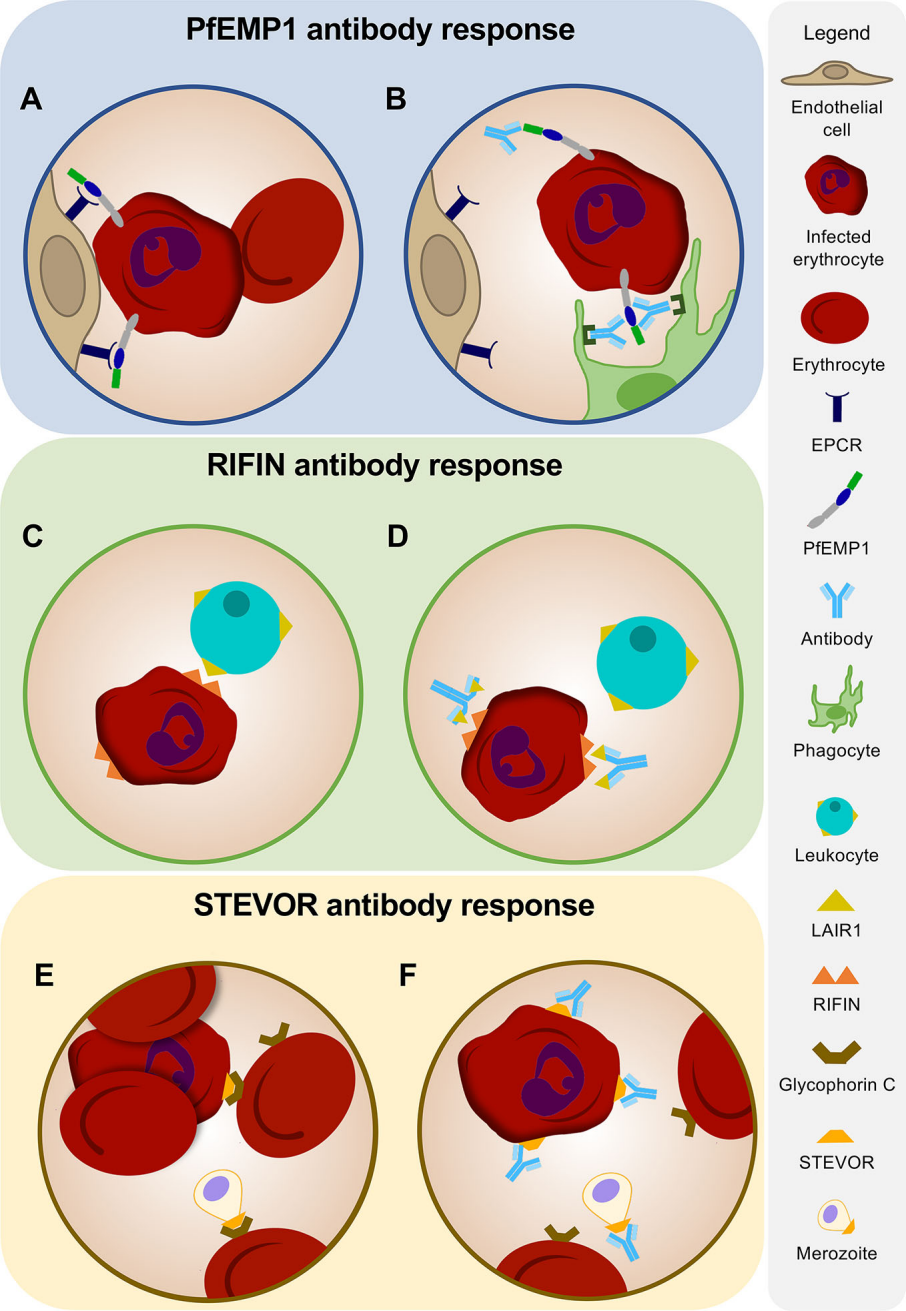
Antibodies against variant surface antigens. **(A)** PfEMP1 on the surface of an infected erythrocyte facilitates binding to receptors on human vascular endothelium. **(B)** Humoral immune responses against PfEMP1 inhibit attachment of infected erythrocytes to host endothelium and induce opsonic phagocytosis of infected erythrocytes. **(C)** Immune evasion of infected erythrocytes is mediated by RIFINs expressed on the surface of the erythrocyte. Binding of RIFINs to LAIR1 on B cells and natural killer (NK) cells results in suppression of immune responses. **(D)** Antibodies against RIFINs may function in preventing the binding of infected erythrocytes to LAIR1 on leukocytes. Broadly reactive antibodies to RIFINs were found to contain an insertion of a fragment of LAIR1 in the arm of the antibody heavy chain. **(E)** STEVORs mediate rosette formation and may play a role in merozoite invasion by adhering to glycophorin C on the erythrocyte surface. **(F)** Antibodies targeting STEVORs can prevent rosetting and inhibit attachment of merozoites to erythrocytes, thereby potentially limiting parasite replication and survival.

During the mature stages of the asexual blood stage, *P. falciparum* expresses variant surface antigens (VSAs) on the cell membrane of the infected erythrocyte. These proteins play crucial roles in both malaria pathogenesis and immune evasion. VSAs belong to multigenic families, most notably *P. falciparum* erythrocyte membrane protein 1 (PfEMP1), repetitive interspersed repeats (RIFIN), and subtelomeric variant open reading frame (STEVOR). PfEMP1 can bind to specific host receptors lining the vascular endothelium, resulting in sequestration of infected erythrocytes in capillaries, thereby preventing parasite clearance by the spleen (16–18) (**Figure 2**). In addition, members of all three VSA families mediate rosetting, the formation of a cluster of uninfected erythrocytes around an infected erythrocyte, which enhances microvascular obstruction (19–22). Rosetting and sequestration of infected erythrocytes can contribute to disease by reducing capillary perfusion and promoting parasite survival by preventing splenic clearance [reviewed in (23)]. Antibodies against VSAs may function by countering these immune evasion strategies of the parasite (24–26). In addition, anti-VSA antibodies may contribute to directly killing infected erythrocytes through the induction of opsonic phagocytosis (27) or antibody-dependent cellular cytotoxicity by natural killer (NK) cells (28).

### Antibody Responses Against PfEMP1

Each *P. falciparum* parasite encodes a diverse set of approximately 60 PfEMP1 proteins, but only one of these antigens will be expressed in a single parasite [reviewed in (29)]. PfEMP1 proteins display a high degree of sequence variation, both among the different variants encoded within a single parasite and among those expressed by different parasite strains. Switching PfEMP1 variant expression therefore contributes to quick and efficient evasion of host humoral immune responses by the parasite (18). Unfortunately, the extreme sequence diversity among PfEMP1 variants has hampered our ability to study naturally acquired immunity against these antigens, as well as the development of a PfEMP1-based vaccine.

PfEMP1 proteins are composed of two to ten cysteine-rich interdomain regions (CIDRα, β, γ, and δ) and Duffy binding-like domains (DBLα, β, γ, δ, ϵ, ζ, and x) that can be further divided into 147 subtypes (30–32). These different domains can bind to different host receptors, including endothelial protein C receptor (EPCR), intercellular adhesion molecule 1 (ICAM1), and CD36. These host endothelial receptors are not expressed at similar levels in all organs (33, 34). For example, CD36 expression is low in brain vascular endothelium, while expression of EPCR is high and expression of ICAM1 is upregulated under conditions of inflammation (33, 34). Pathogenesis, disease symptoms, and the development of severe malaria are therefore partially dependent on which PfEMP1 variant is expressed by the parasite.

Severe malaria is mainly seen in children under 5 years of age and includes life-threatening complications such as cerebral malaria and severe anemia. These severe disease manifestations have been associated with the expression of specific subtypes of CIDR and DBL domains, including CIDRα1, DBLα1, DBLα2, and DBLβ (35, 36). Protection from severe malaria is acquired early in life after only a limited number of infections (37). In the past few years, multiple independent cohort studies have revealed the role of PfEMP1 IgG antibodies in this protective response and have shed light on which PfEMP1 domains are the main targets of protective antibodies. Although all PfEMP1 domains appear to be immunogenic (38), subjects with uncomplicated malaria showed higher anti-PfEMP1 IgG titers and harbored IgGs that target PfEMP1 variants associated with severe malaria (27, 39–42). In a cohort of 448 young children (29–56 months old) in Papua New Guinea, children with uncomplicated malaria had higher levels of anti-PfEMP1 IgGs than children with severe malaria (27). The ability of these antibodies to induce opsonic phagocytosis suggests that direct killing of infected erythrocytes may contribute to protection. However, it remains to be definitively established whether cellular immune mechanisms or the prevention of cytoadherence is the main mechanism of action of these antibodies. Similar to these results, a protein microarray analysis of serum reactivity against 170 PfEMP1 fragments showed that children in Mali with severe malaria had lower PfEMP1 antibody levels and these antibodies recognized fewer PfEMP1 variants than antibodies in control subjects (41). In this study, participants were age and residence matched to minimize differences in parasite exposure between groups. This similarity in exposure levels was experimentally confirmed by the observation that children with cerebral malaria and healthy controls showed only minor differences in immune responses against intracellular PfEMP1 domains and merozoite antigens. However, a limitation of this study is that only PfEMP1 domains from the reference *P. falciparum* strain 3D7 were used, which does not include several known PfEMP1 domains that are associated with severe malaria and does not necessarily match the parasite strains that these children have been exposed to. An extensive study of antibody responses against 456 DBLα domain variants from local parasite isolates among 232 children (0.9–3.2 years old) in Papua New Guinea showed that IgGs against 85 DBLα1 and DBLα2 variants were associated with 70–100% reduction in severe malaria (40). In this study, protection against severe malaria could be further narrowed down to antibody responses against 17 DBLα variants, many of which were physically linked to EPCR-binding CIDRα1 domains (see below). These results suggest that immunity against severe malaria is the result of antibodies targeting a relatively small panel of conserved PfEMP1 domains (less than 5% of the full PfEMP1 repertoire), providing support for developing a vaccine based on these pathogenic PfEMP1 domains. Similarly, Tuju et al. reported that children in Kenya with mild or severe disease (n = 36) showed distinct serological IgG profiles against DBLα domain variants and a similar, but weaker, qualitative difference for IgM (42). The underlying mechanisms that give rise to these different serological outcomes remain to be further investigated and will be informative for vaccine development.

CIDRα1 is a PfEMP1 domain type that may play a dominant role in the development of protection against severe malaria. CIDRα1 domains have been shown to bind to EPCR, and results from several studies suggest that this interaction plays an important role in the development of severe malaria, in particular cerebral malaria (43–45). In cross-sectional studies in two independent cohorts, IgG responses against EPCR-binding CIDRα1 domains were found to develop earlier in life than antibodies against other PfEMP1 CIDR domains (46, 47), highlighting their potential role in protection against clinical disease. In addition, a longitudinal analysis in the Malian cohort showed an ordered acquisition of IgGs against different CIDRα1 domains, starting with IgGs against CIDRα1.7 and CIDRα1.8 (46). This could signify the ordered expression of PfEMP1 variants by *P. falciparum*, potentially in response to the immune status of the host. Alternatively, it could reflect differences in parasite fitness and transmissibility, resulting in early infections preferentially caused by parasites circulating at higher prevalence (46).

Given the high degree of sequence variation among PfEMP1 variants, one would expect that many PfEMP1 antibodies are strain-specific. However, CIDRα1 domains were shown to have conserved structural features that allow many different PfEMP1 variants to bind to the same host receptor (48). This structural conservation of the EPCR binding site may allow the immune system to produce antibodies that are cross-reactive against many different CIDRα1 domains. Indeed, IgGs isolated from serum by affinity purification using a peptide comprising the EPCR binding site of one CIDRα1 domain were able to bind to other CIDRα1 domains and prevented interaction of the domain with EPCR (48). These results suggest that the structural conservation of CIDRα1 domains may provide an opportunity for the development of a vaccine that elicits protection against severe malaria.

### Antibody Responses Against RIFINs

RIFINs are encoded by a set of 150 to 200 genes and are highly antigenically varied (49). Following the pattern of the PfEMP1 family in *P. falciparum*, a single RIFIN is expressed by each parasite (19). Members of the RIFIN family preferably bind to blood group A erythrocytes to form large rosettes and induce cytoadherence, thereby potentially contributing to severe disease (19). In addition, it has recently been shown that RIFINs can bind to inhibitory receptors expressed on B cells, T cells, and NK cells, including leukocyte immunoglobulin-like receptor B1 (LILRB1) and leukocyte-associated immunoglobulin-like receptor 1 (LAIR1) (50) (**Figure 2**). Binding of these receptors to their natural ligands results in the suppression of immune responses (51–53). RIFINs bind the same region of LILRB1 as MHC-I, its natural ligand, and mimic the function of MHC-I by co-localizing with LILRB1 in the immunological synapse and inhibiting NK cell activation (54). In addition, it has been demonstrated that binding of RIFIN to LILRB1 inhibited IgM production in primary human B cells and reduced NK-mediated killing of target cells (55). A direct effect of RIFIN-binding to LAIR1-expressing mouse T cell hybridoma reporter cells has not been observed, but the effect of the interaction between RIFIN and LAIR1 has yet to be tested under more relevant experimental conditions, such as using primary human leukocytes (55). Based on these observations, it has been hypothesized that one of the functions of RIFINs is to down-modulate immune responses against *P. falciparum* through the binding to these inhibitory receptors.

Interestingly, it has been discovered that the human immune system exploits the ability of RIFINs to bind LAIR1 to generate broadly reactive IgM and IgG antibodies to RIFINs (56, 57). These antibodies have highly unusual structures, caused by an insertion of a human LAIR1 exon into the antibody heavy chain variable region CDR3 or into the switch region (**Figure 2**). Gene insertions in switch regions showed higher prevalence in class-switched memory B cells as compared to naive B cells, suggesting a role for activation-induced cytidine deaminase in facilitating such insertions (56, 57). Through somatic hypermutation, these LAIR1 domains lost the ability to bind to their natural ligand, collagen, thereby preventing the generation of auto-antibodies, but retained high affinity for RIFINs. These LAIR1 antibodies were found in 5–10% of malaria-experienced individuals, whereas only 0.3% of European donors had LAIR1-containing IgG (56, 57). However, the presence of LAIR1 antibodies did not correlate with protection against malaria. Thus, while LAIR1 antibodies are testament to the exceptionally strong selection pressure of *P. falciparum* on the immune response of infected individuals, the question remains whether antibodies against RIFINs contribute to protection.

IgG antibodies against RIFINs (irrespective of a LAIR1 insert) were detected in children in Tanzania as early as 1 year after birth, but levels did not increase with age (19). No difference was observed in serum IgG reactivity against three RIFINs on a protein microarray between children (1–6 years old) and adults (18–55 years old) in Mali (58). IgGs in the sera from the adults in this cohort bound to more RIFIN peptides in both semi-conserved and hypervariable domains than did the antibodies in the sera from the children (58). However, there was no difference in seroreactivity between A-type RIFINs, expressed on the surface of the infected erythrocyte, and B-type RIFINs, localized inside the parasite, suggesting that the increased antibody reactivity in adults reflects the higher cumulative exposure to the malaria parasite as compared to the children, but not protection (58, 59). In line with this observation, in children in Kenya, IgGs against PfEMP1 were associated with protection from malaria, while antibodies against other VSAs were not (60), suggesting that PfEMP1 proteins are the main target of protective immune responses against parasite proteins expressed on the erythrocyte surface.

### Antibody Responses Against STEVORs

Of the VSA families discussed in this review, STEVORs were the last to be identified and remain the least characterized. STEVORs represent a family of approximately 40 polymorphic proteins encoded by genes found in subtelomeric regions of the *P. falciparum* genome (61). RNA-sequencing analysis of the *P. falciparum* 3D7 strain revealed a biphasic expression profile of STEVORs within the intraerythrocytic cycle, peaking in early trophozoites and late schizonts/merozoites (62). Additionally, STEVOR expression has been identified in the sporozoite and gametocyte stage of the parasite (63). Localization of STEVORs in all three stages is distinct, suggesting different functions based on the life cycle stage. In both trophozoites and gametocytes, STEVORs can be found on the infected erythrocyte membrane (63), where they likely play a role in parasite sequestration [reviewed in (64, 65)]. In addition, STEVORs have been detected on the apical surface of merozoites (66). STEVORs can bind to glycophorin C on the erythrocyte surface, leading to rosette formation of erythrocytes infected with mature parasites (67) and attachment of merozoite to the erythrocyte membrane (20). Given the widespread distribution of STEVORs throughout the *P. falciparum* life cycle, antibody responses against these targets may inhibit parasite replication at various stages. Indeed, STEVOR-targeting monoclonal antibodies showed a reduction in rosetting (67) and inhibition of merozoite invasion (20).

Although it is clear that STEVORs are involved in host-pathogen interactions, their role in disease pathogenesis is incompletely understood. Several cohort studies have attempted to unravel this role by analyzing serum antibody responses against STEVORs. In a cohort of Ugandan individuals aged 6–22 years old, seroprevalence of antibodies against STEVORs was lower as compared to anti-RIFIN antibodies (68). Grouping of participants based on risk of febrile malaria revealed only a single STEVOR commonly targeted by the humoral immune system of protected individuals (68). In this study, seroreactivity was tested using a library of 30 unique STEVOR recombinant proteins, each expressed as 1 or 2 truncated fragments (68). As a result, it is possible that antibody reactivity was missed because of misfolding of these protein fragments. Future studies will therefore need to evaluate host immune responses to full length STEVOR proteins. A similar study took a closer look at seroreactivity in children versus adults in a Malian cohort using a peptide array (58). Child and adult sera were able to bind all three RIFINs tested in this panel. While adult antibodies also reacted with all six STEVORs, children only showed reactivity to four of the six (58). A peptide array of these six STEVORs revealed that IgG from adults bound significantly more peptides and had more reactivity to semi-conserved and hypervariable regions as compared to IgG from children (58). When comparing sera obtained from children before and 90 days after a single clinical malaria episode, double the amount of STEVOR peptides were recognized by antibodies in the sera at the post-malaria time point, which more closely resembled the epitope profile targeted by adults (58). This study is limited in the repertoire of STEVOR proteins tested and only measured antibody responses against linear epitopes, but it does highlight how quickly antibodies to a single protein can develop over the course of a single infection. These findings are in line with the rapid acquisition of antibodies contributing to protection against severe disease after only a few clinical episodes.

Additional analyses will be required to distinguish if anti-STEVOR antibodies contribute to protection or merely correlate with antigen exposure. Future studies will require a more extensive analysis of STEVOR expression in clinical isolates, similar to what has been accomplished for PfEMP1. The use of 3D7-based antigens to measure anti-STEVOR antibody responses is a limiting factor when trying to understand the contribution of antibody response to protection, since analyses of clinical isolates have revealed regions within STEVORs with high sequence variation (69) and variability in STEVOR expression between fresh isolates and laboratory-adapted strains (66). Using STEVOR sequences from clinical isolates along with cohorts of individuals who are at risk of or clinically protected from malaria will provide a deeper understanding of variants involved in disease pathogenesis, anti-STEVOR antibody responses that are associated with protection, and potential targets for vaccine design.

## *Plasmodium falciparum* Merozoite Antigens

Erythrocyte invasion by merozoites is an essential step in the asexual replication cycle of *Plasmodium* parasites, a process that can be blocked by antibodies [reviewed in (70)]. As a result, merozoite antigens have been the subject of intensive research over the past decades, which unfortunately has not (yet) translated into a successful blood stage vaccine [reviewed in (71)]. Merozoites express a large repertoire of proteins on their surface and in specialized secretory organelles, micronemes, and rhoptries, that are released during the invasion process [reviewed in (70)]. Titers of antibodies against members of the merozoite surface protein (MSP) family are typically among the highest detected in malaria-experienced individuals (72, 73), most likely because of their abundance and exposed nature. However, associations between antibody specificities in serum and protection against malaria are generally weaker for merozoite surface proteins than for microneme or rhoptry proteins (74, 75). Because of genetic polymorphisms and functional redundancies among merozoite proteins, it is still unclear which antigens are the best candidates for vaccine development (8, 9). Delineating the key merozoite antigens and the mechanisms by which antibodies against these antigens confer protection therefore remain important areas of research for the development of a blood stage vaccine.

### Dissecting Protective Antibody Responses to Merozoite Antigens

The prioritization of antigens for vaccine development has been hampered by multiple issues, including our lack of understanding which immunological measures correlate with protection and which mechanisms of antibody-mediated parasite inhibition are most relevant for immunity. Large-scale efforts will therefore be needed to systematically compare antibody responses across different cohorts using multiple readouts. One of the most high-throughput approaches has been to compare antibody specificities between protected and susceptible people using protein microarrays. These analyses have shown that a large proportion of the *P. falciparum* proteome is immunogenic and that the breadth and durability of IgG responses increases with cumulative exposure (5–7). In addition, protected individuals have higher levels of IgG against a broad range of antigens, with unique serological profiles for each individual (5–7). The difference between protection from and susceptibility to malaria cannot simply be explained by the presence or absence of one or a small number of antibody specificities. To better understand the high complexity of such data sets and move beyond analyzing IgG responses to individual antigens, recent studies have used machine learning approaches that are able to assess combinations of responses and their association with protection (76, 77). An analysis of protein microarray data comprising antibody reactivity against more than 1,000 antigens in children (2–10 years old) and young adults (18–25 years old) showed that protection was associated with a qualitative shift in antibody responses, with an increase in antibody titers against VSAs and a decrease in antibody titers against antigens enriched for conserved proteins with unknown function (77). Proietti et al. identified an immune signature of IgG responses against 15 antigens that predicted protection from malaria across two cohorts (76). Interestingly, most of these proteins were localized intracellularly and were expressed at different stages of the *P. falciparum* life cycle, making them unlikely direct targets of parasite-inhibitory antibodies. However, these results may highlight the importance of other components of the adaptive immune system, such as T cell responses, in driving protective B cell immunity. In addition, this immune signature can be used to predict disease susceptibility, monitor the acquisition of naturally acquired immunity over time, and assess the impact of malaria interventions.

One shortcoming of these large-scale protein microarray studies is that they only assess antibody binding, not antibody effector functions such as opsonization or complement fixation. Antibodies that bind to merozoite antigens do not necessarily inhibit erythrocyte invasion. For example, it has been shown that the activity of inhibitory anti-MSP1 IgG can be blocked by non-inhibitory antibodies that compete for binding to overlapping epitopes or bind to more distal regions and presumably prevent binding of inhibitory antibodies through steric hindrance (78). Assays that assess different antibody effector functions are therefore essential to measure the ability of antibodies to inhibit parasite replication or induce parasite killing by immune cells. In recent years, the importance of antibody effector functions in parasite inhibition has been demonstrated and several studies have started to delineate the contribution of antibodies to individual antigens in this process. In the following sections, we summarize recent advances in our understanding of the nature, and mechanisms of acquired antibody-mediated protection against *P. falciparum* merozoite invasion.

### Antibody Responses Against Combinations of Merozoite Antigens

Recent studies on naturally acquired immunity have provided the important insight that the breadth of antigen recognition is more predictive of immunity than antibody reactivity against any single antigen (74, 75, 79–82). This would suggest that a vaccine based on a single antigen may not be successful in eliciting a fully protective antibody response. To better understand how many different antigens should be targeted to achieve protection and whether antibody responses against certain combinations of antigens perform better than others, the cumulative protective effect of individual antibody specificities has been analyzed. In both Sub-Saharan African and Indian populations, combinations of IgGs against four or five antigens provided nearly full protection against malaria, with only limited contributions from antibodies against additional antigens (80, 82). The most effective combinations were reported to target antigens presented during distinct stages of the invasion process, thereby working synergistically to inhibit invasion (79). In some of these combinatorial analyses, IgG responses against several uncharacterized proteins that do not have a functional annotation but are referred to by their gene identifiers (for example, PF3D7_1136200 and PF3D7_0606800) were associated with the highest levels of protection. These findings support the approach to develop a multi-antigen vaccine and encourage further evaluation of several novel protective antigens as potential vaccine candidates.

### Strain-Transcending Immunity

Circulating *P. falciparum* strains are genetically diverse (8, 9). One of the challenges for vaccine development is to elicit immunity against these genetically distinct parasite strains. Passive immunization studies in the 1960s and 1990s showed that the transfer of IgG from immune individuals living in West Africa to malaria patients in East Africa or Thailand resulted in almost complete clearance of parasitemia in the absence of other treatment (83, 84). These results suggest that IgG from immune individuals can inhibit the replication of different *P. falciparum* strains from diverse geographical regions. However, the question remained whether this protective effect was the result of a combination of strain-specific antibodies targeting multiple genetically diverse antigens, or if this was due to one or more antibodies targeting conserved epitopes and thereby conferring strain-transcending immunity. A recent study by Hill et al. sought to answer this question by measuring opsonic phagocytosis of merozoites from 15 different parasite strains induced by serum antibodies from semi-immune children (5–14 years old) living in Papua New Guinea (85). Children with serum IgGs that promoted phagocytosis of merozoites from all strains tested showed an 85% decrease in the risk of developing malaria during 6 months of follow-up as compared to children with narrow opsonic phagocytosis activity in serum. Depletion of serum antibodies that bound to one merozoite strain drastically reduced opsonization of merozoites from other *P. falciparum* strains. These results suggest that opsonic phagocytosis activity against merozoites from different strains is mediated by the same antibodies. However, it is important to mention that *P. falciparum* exposure levels were not matched between groups and it can therefore not be ruled out that differences in opsonic phagocytosis activity between these groups were driven by differences in antibody titers. More research will be required to fully understand the dynamics of strain-transcending immunity and to identify the conserved epitopes targeted by this response.

### Antibody Effector Functions Associated With Protection

One of the priorities for malaria vaccine development is the establishment of correlates of protection and the standardization of *in vitro* assays to measure these immunological parameters. It is therefore important to better understand the molecular and immunological mechanisms by which antibodies inhibit merozoite invasion. In general, antibodies can act by blocking interactions between molecules (i.e., neutralization), or by promoting Fc-mediated effector functions, including complement activation, opsonic phagocytosis, and antibody-dependent cellular cytotoxicity.

**Figure 3:**
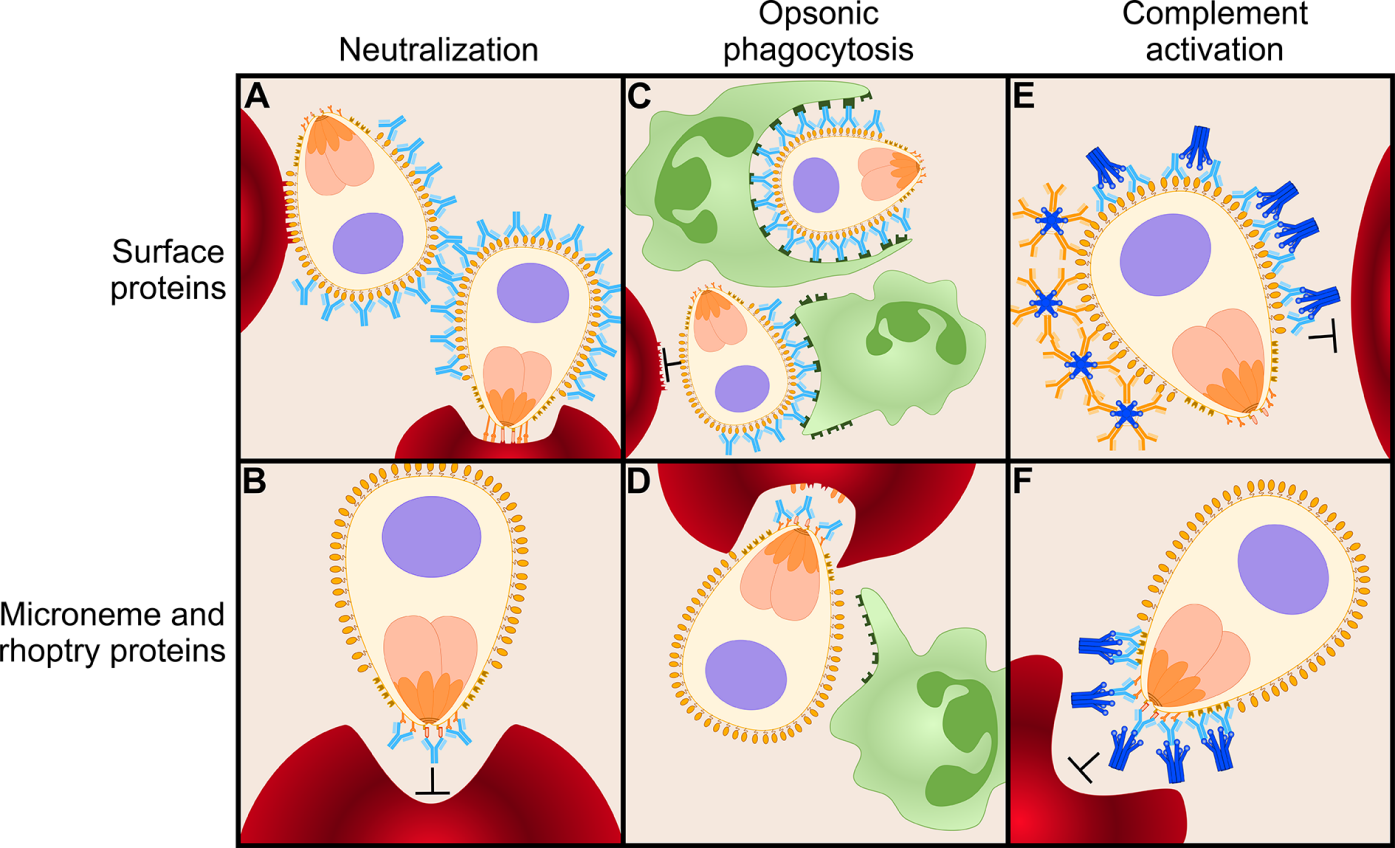
Antibody-mediated inhibition of merozoites. Antibodies can function in several ways to mediate inhibition of merozoite invasion into erythrocytes. Here, we present a model of various antibody effector mechanisms for two different classes of antigens: highly abundant merozoite surface proteins and relatively scarce microneme and rhoptry proteins. **(A)** Immunoglobulins G (IgGs; shown in light blue) targeting surface proteins are not effective mediators of inhibition *via* neutralization, possibly because extremely high antibody concentrations are necessary to completely block the coat of proteins on the merozoite surface. **(B)** IgGs can effectively neutralize merozoites when targeting relatively scarce microneme and rhoptry proteins on the apical surface, such as PfRH5 and EBA-175. **(C)** When targeting abundant surface proteins, opsonic phagocytosis is effectively mediated *via* IgG1 and IgG3 by binding of phagocytic cells (green) to their Fc domain. **(D)** Many microneme and rhoptry proteins are only released to the surface of the merozoite once it has attached to an erythrocyte and is ready to commence the invasion process. At this moment, the erythrocyte will (partially) shield IgGs bound to microneme and rhoptry proteins from the Fc receptors of phagocytes. Thus, opsonic phagocytosis may not be an efficient effector function for antibodies against this class of proteins. **(E)** Both IgGs and IgMs (orange) efficiently initiate the classical complement cascade *via* C1q (dark blue) when targeting surface proteins to prevent merozoite invasion. **(F)** IgG is an effective mediator of the complement cascade when targeting microneme and rhoptry proteins. IgM may be less efficient against this class of proteins because large, pentameric IgM may not be able to reach between the erythrocyte and merozoite surfaces.

Antibodies against several merozoite antigens have been shown to act by neutralization. For example, IgG binding to EBA-175 prevented attachment of this protein to glycophorin A on the erythrocyte surface, thereby inhibiting invasion (86). Similarly, anti-PfRH5 IgG engineered to not engage in any Fc-mediated effector functions was able to protect against *P. falciparum* challenge, demonstrating that neutralization is likely to be the primary mechanism of action (87). Although the growth inhibition assay has traditionally been the gold standard *in vitro* assessment of antibody neutralization, it generally does not correlate well with naturally acquired protection against malaria and may therefore be considered less valuable for assessing naturally acquired immune responses than other, more recently developed, assays (74). However, the growth inhibition assay seems well suited to measure the activity of antibodies that are known to mediate their effect through neutralization. We speculate that these antibodies most likely target antigens that are less abundant on the merozoite surface and are involved in essential interactions with ligands on the erythrocyte (**Figure 3**). The growth inhibition assay may therefore be the assay of choice to evaluate vaccine-elicited antibody responses against EBA-175, PfRH5, and potentially other antigens.

The implementation of novel functional assays has revealed the role of opsonic phagocytosis and complement fixation in antibody-mediated inhibition and has uncovered these effector functions as correlates of naturally acquired protection. As mentioned, opsonic phagocytosis by naturally acquired antibodies, mainly IgG1 and IgG3, has been reported to be strain-transcending (85) and correlates well with protection from disease (81, 88). Opsonic phagocytosis of whole merozoites increased with greater breadth of antibody responses (81, 88), confirming that targeting multiple antigens is beneficial for anti-merozoite immunity. Various antigens have been identified as targets for these opsonizing antibodies, including MSP1, MSP2, and MSP3 (86, 88, 89). To further delineate the role of individual antigen specificities, Kana et al. used beads coated with recombinant proteins to measure the ability of IgG antibodies against single merozoite antigens to induce opsonic phagocytosis (90). Antibody-induced phagocytosis in this assay showed an association with protection against malaria in a longitudinal cohort of 134 children in Ghana for MSP1-19, MSP2 (3D7 allele), MSP3 (N-terminal region of K1 allele), GLURP (region 2), and PfRH2-2030 (the fragment of PfRH2 covering amino acid residues 2030–2528), but not against 21 other antigens that have been implicated as targets of the protective immune response (90). Combinations of antibody responses against six antigens correlated better with protection than responses to any individual antigen, suggesting that antibodies against multiple antigens may be necessary to induce optimal merozoite phagocytosis.

Complement fixation and subsequent activation of the classical complement pathway is also strongly associated with protection for various merozoite antigens (74, 91). This complement cascade culminates in the formation of the membrane attack complex, which mediates merozoite lysis. Reiling et al. determined that fixation of C1q, the first step in the complement cascade, correlated strongly with membrane attack complex formation and therefore measured the ability of serum IgM and IgG antibodies against various merozoite antigens to bind C1q as a proxy of complement-activating potential (74). The prevalence of C1q-fixing antibodies was high for the majority of merozoite antigens studied and correlated strongly with protection against malaria. The strongest protective associations were observed for C1q-fixing antibodies against EBA-140 (region III–V), MSP7, rhoptry-associated leucine zipper-like protein 1 (RALP1), GPI-anchored micronemal antigen (GAMA), and PfRH2-2030 (74). Again, combinations of antibodies against three different antigens showed an increased protective effect over single antigens or combinations of two antigens, with RALP1 and MSP7 among the antigens most frequently found in protective combinations.

Interestingly, strong opsonic phagocytosis activity was mainly observed for antibodies targeting antigens expressed on the merozoite surface, while the strong complement-fixing activity was predominantly observed for antibodies directed against rhoptry and microneme proteins (74, 90). Of the antigens that were tested in both assays, antibodies that displayed strong opsonic phagocytosis activity against three out of four antigens in the bead-based assay performed by Kana et al. showed relatively weak or no complement-fixing activity and the reverse was true for IgG antibodies against EBA140RIII-V and RALP1 (74, 90). It is likely that multiple antibody effector mechanisms act in concert to provide immunity (92). Both complement fixation and opsonic phagocytosis are induced by the cytophilic IgG1 and IgG3 antibodies, underlining that a vaccine-induced response against merozoite antigens should be directed toward these isotypes. The results from Reiling et al. and Kana et al. suggest that cytophilic antibodies against different antigens preferentially act through one particular effector function, and it will be interesting to understand which antigenic properties influence this response. One explanation for the preferential opsonic phagocytosis activity of antibodies against merozoite surface proteins could be that many antigens located in rhoptries and micronemes only become exposed when the merozoite is in direct contact with an erythrocyte (93) [reviewed in (70, 94)] and may therefore be poorly accessible to phagocytes (**Figure 3**). However, the differences between results from Reiling et al. and Kana et al. could also be influenced by variation in antibody titers between individuals, the studied antigens, characteristics of the cohort, inclusion criteria for participants selected for the study, as well as other variables. To further evaluate the contribution of antibody specificities and effector mechanisms to inhibition of merozoite invasion, samples from the same individuals should be compared side-by-side in assays that measure these different effector functions against the same panel of merozoite antigens. Epitope mapping of antibodies with different levels of inhibitory activity against merozoites will also provide important insights into immune mechanisms. For example, binding of monoclonal IgGs to a non-neutralizing epitope of merozoite protein PfRH5 resulted in slower erythrocyte invasion, thereby increasing exposure times of other epitopes on PfRH5 to neutralizing antibodies (95). This resulted in a synergistic effect between the non-neutralizing and neutralizing antibodies (95). For MSP1, epitope mapping of non-inhibitory and inhibitory antibodies revealed MSP1 cleavage sites as the most vulnerable sites of this antigen (96). It would be highly informative to also perform such studies for other antigens to better understand how antibodies function and which epitopes should be targeted by vaccination to achieve an effective immune response.

### Immunoglobulin M Responses Against Merozoite Proteins

Although the focus of studies on naturally acquired immunity has been on IgG, recent studies suggest that IgM may also play an important role in protection (97–99). IgM is an excellent mediator of complement activation and may function in this way to confer immunity (98). An interesting observation about the role of IgM in protection against malaria has come from a comparison between the Fulani and Dogon people, two genetically distinct ethnic groups living in West Africa with different susceptibility to malaria (100). The Fulani are more resistant to malaria but carry lower frequencies of classical malaria-resistance genes (101), which has prompted a search for immunological variables that can explain the observed differences in malaria susceptibility. Differences in the frequency of genetic variants in several immune-related genes have been identified between the Fulani and Dogon people, but the immunological consequences of these results are unclear (102). Transcriptional profiling showed that the Fulani have a strong inflammatory gene signature in the monocyte population during *P. falciparum* infection, leading to the hypothesis that monocytes in Fulani people exist in a “primed” state that will elicit a stronger immune response upon infection (103). The Fulani also had higher frequencies of activated memory B cells in the absence of infection and higher percentages of plasma cells during *P. falciparum* infection (104). These enhanced cellular immune responses most likely explain the increased IgM and IgG levels against many *P. falciparum* proteins in the Fulani as compared to the Dogon (97). Interestingly, the Fulani IgM responses recognized a broader repertoire of *P. falciparum* proteins than IgG responses, including a set of antigens to which antibody levels have been correlated with protection (97). These results suggest that IgM may play an underappreciated role in protection against malaria.

In a mouse model, IgM^+^ memory B cells were shown to be the first responders in secondary *Plasmodium* infections, giving rise to both IgM and IgG antibody-secreting cells (99). IgM^+^ memory B cells contained somatic hypermutations and recognized target antigens with high affinity, pointing to T-cell dependent affinity maturation within the germinal center. IgM^+^ memory B cells with specificity for merozoite antigens were also detected in malaria-experienced humans (99). Finally, Boyle et al. recently showed that IgM against merozoite antigens appears rapidly upon *P. falciparum* infection and reaches high levels in individuals with life-long exposure (98). IgM levels were more stable under low transmission conditions than IgG levels and were associated with protection (98). However, children with high IgM levels also had high levels of IgG against merozoite antigens, which were also associated with protection. The independent contribution of these antibody classes to immunity therefore remains to be determined.

## The Acquisition and Maintenance of Humoral Immune Responses Against *Plasmodium falciparum*

Naturally acquired antibody responses against *P. falciparum* require repeated parasite exposure to attain protection, while probably never reaching sterilizing immunity. The rate of antibody acquisition against *P. falciparum* proteins is influenced by various factors, including age of the human host, transmission intensity, and the type of antigen (72, 73, 105, 106). In general, antibody levels increase with both age and higher transmission intensity (72, 73, 105). Antibodies against different antigens accumulate at varying rates, with stronger responses typically seen for antibodies targeting extracellular or plasma membrane proteins and proteins that are highly abundant, highly polymorphic, or lack a human ortholog (106).

It has been shown that IgGs against PfEMP1 are acquired during the first years of life (47, 107), and their breadth increases with age and exposure (38, 60). In a cohort of children from Papua New Guinea, levels of PfEMP1 IgG antibodies positively correlated with protection in both younger children (1–4 years) and older children (5–14 years) (107). Interestingly, antibodies against merozoite antigens were only associated with protection in older children in this cohort (107). These results suggest that antibodies against PfEMP1 may provide a first line of defense against severe malaria pathogenesis early in life, while antibodies to other parasite antigens may contribute to protection against uncomplicated malaria in older children. This may seem counterintuitive given the extremely high diversity among PfEMP1 variants, at the level of both amino acid sequences and domain composition, while polymorphisms in merozoite antigens provide relatively modest sequence variation in comparison. However, as detailed in Section 2.1, only a small fraction of PfEMP1 domains are pathogenic, with particularly strong evidence for the role of CIDRα1 in severe malaria. Targeting one or a small panel of such pathogenic PfEMP1 domains may be sufficient to achieve protection against severe disease. In contrast, a broader antibody response with higher antibody titers may be necessary to effectively inhibit erythrocyte invasion by merozoites. Indeed, it has been reported that antibodies against merozoite antigens must reach a threshold level before associating with protection (10, 11). While such a functional threshold level is also likely to exist for antibodies against PfEMP1 and antigens on other pathogens, the requirement for recognition of multiple merozoite antigens at high antibody concentrations may be exceptionally high for *Plasmodium* infections. This may explain why relatively low antibody levels against merozoites in young children are not sufficient to provide protection to malaria in these individuals, while similar levels of anti-PfEMP1 antibodies confer protection against severe disease. The contribution of immune responses against these two main groups of antigens to immunity remains to be fully untangled.

An additional complication to the generation of a protective antibody response against *P. falciparum* is the ability of the parasite to modulate immune responses *via* several mechanisms. *Plasmodium* infections induce a pro-inflammatory immune response that drives differentiation of CD4^+^ T cells into Th-1 polarized T follicular helper cells, resulting in impaired germinal center (GC) responses (108, 109). This pro-inflammatory environment also induces the expansion of a subset of B cells known as “atypical memory B cells” (110, 111) [reviewed in (112, 113)]. These cells express inhibitory receptors and have been described as functionally impaired (114, 115), although several studies have reported that these cells may in fact be functional and can contribute to anti-parasite immunity (116, 117). It has been postulated that naive B cells differentiate into atypical memory B cells in B cell follicles upon stimulation with interferon gamma produced by Th-1 polarized T follicular helper cells and Toll-like receptor agonists (118). In this process, atypical memory B cells may bypass the germinal center (GC) or exit the GC prematurely, thus limiting affinity maturation and the ability of these cells to produce high affinity antibodies. Finally, a recent study showed that in both human and mouse experimental *Plasmodium* infections, a large proportion of activated B cells differentiated into extrafollicular plasmablasts (119). The high demand of replicating plasmablasts for nutrients, in particular the amino acid L-glutamine, outcompeted that of GC B cells, thereby limiting the development of memory B cells and long-lived plasma cells. The disruption of GC formation during *Plasmodium* infections likely hampers the development of B cells with high affinity for parasite antigens. It is however conceivable that the chronic and repetitive nature of *P. falciparum* infections will ultimately allow for the acquisition of high levels of somatic hypermutations by consecutive rounds of memory B cell activation and affinity maturation, as observed in, for example, HIV-1-infected individuals (120–122). Alternatively, high affinity plasma cells could be derived from naive B cell precursors with intrinsic high binding affinity, which would limit the need for effective germinal center reactions (123). Longitudinal molecular studies of antibody evolution have not been performed in naturally *P. falciparum*-exposed individuals but would be useful to better understand how protective antibody responses develop over time.

Given the high levels of antibodies required for protection against malaria, protective antibody responses are generally not sustained over long periods of time without re-exposure. Antibody levels are maintained by long-lived antibody-secreting cells in the bone marrow. While these plasma cells are not easily accessible for study in humans, their half-life can be modeled based on serum antibody levels. Using this approach, Yman et al. found that antibody levels against merozoite antigens as well as the proportion of long-lived antibody-secreting cells after a malaria episode in individuals who were formerly exposed because of residency in an endemic area (a median of 14 years ago) were higher compared to previously malaria-naive travelers (12). These results suggest that repeated *P. falciparum* infections are necessary to drive the formation of long-lived antibody-secreting cells, providing an explanation for the observation that immunity requires prolonged exposure. In addition, the half-life of merozoite antigen-specific long-lived plasma cells was 2–4 years, which was shorter than that of long-lived antibody-secreting cells against tetanus toxoid (7 years) and explains why some degree of exposure is necessary to maintain immunity (12). However, this study also demonstrated that immunological memory is not completely lost after more than a decade of non-exposure. These results are in agreement with an earlier report that showed that in five Swedish residents who were previously treated for malaria following international travel, *P. falciparum* merozoite antigen-specific memory B cells were detected up to 16 years later in the absence of re-exposure (124). Findings from a recent study in a low transmission region in Zambia suggest that the level of exposure necessary to maintain antibody levels is probably very low (73). In this setting, parasite prevalence in the population was approximately 2.5%, most infections were asymptomatic, and about half of all individuals with parasitemia had parasite levels that were undetectable with rapid diagnostic tests. In the absence of significant recent transmission, adults showed stable antibody levels over a period of 2 years, suggesting that infrequent infections with low-level parasitemia are sufficient to maintain humoral immune responses. Among targets of the strongest antibody responses were PfEMP1 variants and merozoite antigens. Neither of the studies discussed above included a functional assessment of antibody responses, and it is thus unclear whether the antibodies measured in these studies contribute to protection. However, assuming that long-lived antibody-secreting cells and antibodies targeting protective epitopes follow similar kinetics, these results suggest that long-term protection by vaccination may be possible with frequent boosting of the humoral immune response.

## Concluding Remarks

The main targets of naturally acquired immune responses against *P. falciparum* are variant surface antigens on the plasma membrane of the infected erythrocytes and antigens expressed by merozoites. Antibody responses against PfEMP1 variants develop early in human life and seem to be the main driver of protection against severe malaria in young children. Protection against malaria in older children is achieved once antibodies against merozoite antigens reach sufficiently high levels. A vaccine based on a combination of these two classes of antigens may elicit antibodies that have an additive effect, or possibly even act synergistically, toward the prevention of all clinical manifestations of malaria.

The main obstacle for the development of a vaccine targeting PfEMP1 is the identification of conserved epitopes, given the enormous sequence variation among this protein family. The CIDRα1 domain has the most potential as a vaccine candidate against severe malaria, given its role in the pathogenesis of severe malaria through binding to EPCR, and because of structural conservation of the EPCR binding site.

Correlations with protection have been observed for antibody responses against many different merozoite antigens. Broader antibody responses provide better protection, with near full protection for responses against combinations of three to four antigens. What remains unclear is which of these (combinations of) antigens should be moved forward for vaccine development. Since many of these antigens are polymorphic, the identification of conserved epitopes has high priority. In addition, several candidates that have been identified have unknown functions and further characterization of these proteins will provide more insight into the merozoite invasion process.

Finally, recent studies have revealed mechanisms of antibody-mediated parasite inhibition. Understanding how antibodies function against different target antigens is important to be able to induce the most effective immune response by vaccination and to establish correlates of protection. Nature has provided us with proof-of-concept that protection against blood stage infection is achievable. Our increased understanding of naturally acquired immunity therefore provides hope for the development of a malaria blood stage vaccine.

## Author Contributions

The first draft of the manuscript was prepared by SG and RR, and revised by EB. AB, SB, and GB further edited the manuscript and provided intellectual contributions to the design of the figures. All authors contributed to the article and approved the submitted version.

## Funding

This work was supported by the National Institutes of Health [grant numbers AI128466 and AI133274]. SG and AB are supported by Graduate Research in Immunology Program training grant NIH T32 AI138944. RR is supported by the National Center for Advancing Translational Sciences of the National Institutes of Health under Award Number TL1TR002647.

## Conflict of Interest

The authors declare that the research was conducted in the absence of any commercial or financial relationships that could be construed as a potential conflict of interest.
